# Comparison of clinical and radiologic treatment outcomes of Kienböck’s disease

**DOI:** 10.1186/s13018-015-0276-7

**Published:** 2015-08-27

**Authors:** Stéphane Stahl, Pascal J. H. Hentschel, Adelana Santos Stahl, Christoph Meisner, Hans-Eberhard Schaller, Theodora Manoli

**Affiliations:** Department of Plastic, Hand and Reconstructive Surgery, Burn Center, BG-Trauma Center, Eberhard-Karl University, Schnarrenbergstr. 95, 72076 Tübingen, Germany; Department for Plastic Surgery, Marienhospital Stuttgart, Böheimstr. 37, 70199 Stuttgart, Germany; Institute for Clinical Epidemiology and Applied Biometry, Eberhard-Karl University of Tübingen, Silcherstr. 5, 9572076 Tübingen, Germany

**Keywords:** Lunate necrosis, Kienböck’s disease, Osteonecrosis, Kienböck, Case control study, Radial shortening osteotomy, Vascularized bone graft, Scaphotrapeziotrapezoid arthrodesis

## Abstract

**Purpose:**

The clinical outcomes of scaphotrapeziotrapezoid (STT) arthrodesis were compared to radial shortening osteotomy (RSO) to determine if any of the treatment methods was superior. The impact of RSO and vascularized bone grafts (VBG) on disease progression were measured based on X-rays to evaluate if a difference in Kienböck’s disease (KD) progression exists.

**Methods:**

Out of 98 consecutive patients treated between 1991 and 2013, 46 had STT arthrodesis, 21 had RSO, 7 had VBG, and 3 had VBG and RSO. Patients treated with STT arthrodesis were compared to RSO regarding post-operative range of motion (ROM), wrist pain on the Numeric Rating Scale (NRS), grip strength, duration of incapacity for work, the Disabilities of the Arm, Shoulder, and Hand (DASH), and the Modified Mayo Wrist scores (MMWS). Radiographic assessment (Nattrass index, radioscaphoid angle, and Ståhl index) was performed to determine disease progression following RSO or VBG. Baseline patient characteristics were comparable in all treatment groups.

**Results:**

There were no significant differences in post-operative ROM, wrist pain, grip strength, duration of incapacity, DASH score, or MMWS score following STT arthrodesis (*n* = 27) or RSO (*n* = 14). The Ståhl index, the Nattrass index, and the radioscaphoid angle suggested disease progression following RSO (*n* = 14) and/or VBG (*n* = 6) although the changes were not significant.

**Conclusions:**

The study failed to demonstrate clinically relevant differences between STT arthrodesis compared to RSO. No evidence was found that decompression or revascularization, or the combination of the two, can reverse or halt the course of the disease.

**Level of evidence:**

Therapy, level III, retrospective comparative study with prospectively collected data.

## Introduction

Treatments for Kienböck’s disease (KD) can be grouped into three categories: symptomatic, salvage, and causal. Symptomatic treatment like wrist denervation is expected to decrease pain, and salvage procedures like scaphotrapeziotrapezoid (STT) arthrodesis are anticipated to prevent the onset of arthritis thereby prolonging wrist function in time, while causal treatments like radial shortening osteotomy (RSO) and/or vascularized bone grafts (VBG) are thought to halt or reverse the progress of the disease.

Recent case–control studies [1, 2], systematic reviews [2, 3], and a meta-analysis [2] have shown that neither high-level nor good quality scientific evidence exists to support any of the hypotheses regarding the etiology of KD published in the literature. Therefore, causal treatment of KD has to be critically evaluated on the basis of evidence of restored trabecular architecture, lunate shape and wrist geometry (effectiveness), and a superior success rate compared to other treatments in comparative studies (efficacy).

In a systematic review of 205 articles on the treatment of KD, salvage and causal treatment options presented similar positive outcomes [4]. However, comparisons across treatments were not possible. Surgical treatments of rare diseases usually lack controlled trials and formal statistical analyses; thus, physicians base their clinical judgments solely on potentially biased observational studies, experience, or anecdote [5]. Few studies present objective and adequate parameters to verify if the surgical treatment achieved the goal for which it was indicated [4], and even fewer studies compare the outcomes of two different procedures.

The purpose of this study was to perform a comparative analysis of the clinical outcome of STT arthrodesis vs. RSO using a standardized self-assessment questionnaire, a face-to-face interview and clinical measurements to determine if one treatment method was more effective than another. Furthermore, the pre- and post-operative radiological parameters associated with RSO and VBG were compared by three independent surgeons to determine if their impact on disease progression differed.

## Patients and methods

### Patients

The study was conducted with the approval of the Ethics Review Board of Eberhard-Karls-University, Tuebingen, Germany (approval number 176/2009BO1).

Between January 1990 and October 2013, 98 consecutive patients, treated for the first time for KD, were identified based on an electronic archive of a large teaching hospital (of more than 1800 beds) certified as a level-I trauma center. All patients were included who had a documented clinical examination, a standardized pre- and post-treatment X-ray examination, a pre-treatment CT scan and MRI exam with contrast agents, and a follow-up period of at least 6 months. In addition, all patients who provided written informed consent and completed a case report form were included in the study. The case report form consisted of a medical record evaluation, a self-assessment questionnaire, an interview and clinical examination, and a standardized radiological assessment form. We excluded all patients with an incomplete case report form. All follow-up examinations were performed in the same hospital by an independent surgeon not otherwise involved in the study.

Out of the 98 patients treated for KD between 1990 and 2013, 85 % had complete medical data. In 73 % of the cases, complete questionnaires were returned. Ninety-three percent of these patients had a clinical and radiological follow-up examination. In total, 26/46 patients after STT arthrodesis, 14/21 patients after RSO, 4 out of 7 patients after VBG, and 2 out of 3 patients after VBG and RSO were available for final review with complete clinical and radiological data.

The operative techniques were performed as previously described (STT arthrodesis without lunate resection [6], RSO [7], vascularized bone grafts (VBG) of the 4th extensor compartment artery [8], and palmar VBG [9]). Post-operative cast immobilization was maintained for 6 weeks. All surgeries were performed by either one of five plastic surgery-trained hand surgeons or senior resident/fellow under direct supervision from a faculty member at one single institution. The treatment recommendations for STT arthrodesis, RSO, and VBG were based on previously published expert opinion [10]. Wrist denervation was not performed as an adjunct to the above surgeries but as a distinct primary or secondary procedure in patients excluded from this analysis.

In all cases, the pre-operative diagnosis was confirmed by one of five plastic surgery-trained hand surgeons and a consultant radiologist with expertise in musculoskeletal radiology. Patients were grouped according to the type of treatment administered (i.e., RSO or STT) (Table 1). In three patients, RSO was performed in stage IV as a last resort because the patients did not consent to total wrist arthrodesis. In these three cases, arthritis was limited to lunate cartilage.

**Table 1:** Baseline characteristics of all treatment groups

Type of treatment		Patients operated	Patients at follow-up	Follow-up in years		Age at treatment	Dominant hand affected	Employment status		Occupational group	
								Full-time work^a^	Unemployed^b^	Blue collar	White collar
		Total	Total	Mean	SD	Mean	No. (%)	No. (%)	No. (%)	No. (%)	No. (%)
STT		46	27	4	2.99	37	19 (70)	25 (93)	2 (7)	12 (44)	13 (48)
RSO		21	14	10	7.43	34	9 (64)	12 (86)	2 (14)	4 (29)	8 (57)
VBG^c^		7	4	1	0.7	38	1 (75)	4 (100)	0	2 (50)	2 (50)
VBG^d^ and RSO		3	2	4	1.06	29	1 (50)	2 (100)	0	2 (100)	0
Differences											
STT vs. RSO	*p*			<0.01^e^		0.87^e^	0.69^f^	0.54^f^	0.49^f^	0.28^f^	0.59^f^

*STT* scaphotrapeziotrapezoid arthrodesis, *RSO* radial shortening osteotomy, *VBG* vascularized bone graft, *SD* standard deviation

^a^Full-time work or part-time work (including training/professional training)

^b^Maternity, unemployed for at most 12 months, or never been employed

^c^Six palmar and one dorsal VBGs

^d^One dorsal and two palmar VBGs

^e^ *t* test

^f^Fisher exact-test

### Medical record evaluation

Data retrieved from the standardized medical records included the dates of all examinations, the date and type of treatment, secondary treatments and complications, pre-treatment active range of motion (ROM), duration of wrist immobilization, incapacity for work, and epidemiological data (e.g., age, gender, and handedness). Data were retrieved from the paper-based and electronic patient records to compensate for missing data. Inconsistencies were resolved during the interview. The accuracy and completeness of the case report form was verified on the occasion of the clinical examination to avoid missing data and to exclude misunderstandings.

### Self-assessment questionnaire

The patients were invited for a follow-up examination, and a questionnaire was delivered by mail and was re-sent 6 weeks later to non-responders. Dillman’s total design method (introductory letter, a self-assessment questionnaire, and an informed consent form with a stamped return envelope) was used to maximize response rates [11]. If no response was received in the following 6 weeks, the patients were contacted by telephone, and an appointment for the clinical examination was arranged during which the self-assessment questionnaire was given to the patient. The questionnaire was developed by a multidisciplinary team consisting of one occupational physician, two hand surgeons, one psychiatrist, and one epidemiologist, as previously described [1].

Besides demographic, occupational, and medical items, the questionnaire contained the functional outcome parameters: the Disabilities of the Arm, Shoulder, and Hand (DASH) score including the optional work module, the Modified Mayo Wrist Score (MMWS). The query as to whether or not the patient would have the same operation if he/she were given the same choice, requiring a simple “yes or no” response and duration of work incapacity were also included.

Pain was assessed using a Numeric Rating Scale for Pain (NRS) on an 11-point numeric scale with “0” representing no pain and “10” representing the worst pain imaginable at either rest or activity-induced [12].

### Interview and clinical examination

The standardized interview assessed duration of work incapacity, current medication, prior surgery, and medical history. The standardized clinical examination included active ROM in extension/flexion (E/F), radial/ulnar (R/U) deviation, pronation/supination (P/S) as measured with a conventional goniometer, grip strength as measured with a Biometrics® dynamometer, and tenderness and signs of accompanying diseases of the hand [13]. The grip strength ratio was calculated by dividing the grip strength in the KD wrist by the contralateral side to exclude personal factors influencing grip strength (age and gender biases). To compensate for the effect of hand dominance, two ratios were calculated: one ratio when KD affected the non-dominant side: non-dominant/dominant side and another ratio when KD affected the dominant side: dominant/non-dominant side.

### Radiological assessment

Pre-treatment imaging (including X-ray examination of both wrists and CT and MRI scans) was retrieved from the hospital’s digital and conventional X-ray archive, or the referring surgeons, if necessary. Radiological assessment included pre- and post-treatment Ståhl index (lunate height on lateral view/lunate width on lateral view) [14], Nattrass index (carpal height on PA view/capitate height on PA view) [15], radioscaphoid angle using the tangential method [16], and disease stage according to Lichtman [17]. Stage IIIB was defined as a radioscaphoid angle greater than 60° [18]. Stage IV was defined as the presence of any sign of cartilage damage on the lunate or the lunate facet on X-ray (joint space narrowing, irregular joint margin, osteophyte formation, cyst formation, or subchondral sclerosis). Indices and angles were measured, independently, by three independent board certified plastic surgeons (hand fellows) using standardized X-rays. A radiological assessment form, with instructions and line drawings from the above references, was given to all examiners. Disagreements were resolved through consensus. Because STT arthrodesis provides a stable framework and mechanism for load transference through the wrist via the capitoscaphoid and radioscaphoid joints, thereby, maintaining the carpal height index stable irrespective of the course of progression of KD, radiological progression was not assessed after STT arthrodesis [19].

### Statistical analysis

Differences among outcomes were analyzed with the Fisher’s exact, Wilcoxon test, or the *t* test, where appropriate. *P* values <0.05 were accepted as statistically significant without adjustments for multiple comparisons. A retrospective power analysis was performed to determine if the sample size of our study was adequate to make comparisons between treatment groups. Power was calculated according to the method of Cohen (sample size calculation in last line of Tables 2, 3, and 4). SPSS Version 17 (SPSS Inc., Chicago, IL, USA), SAS 9.2 (SAS Institute Inc. Cary, NC, USA), and SPSS 21 (IBM Corp, Released 2012, IBM SPSS Statistics for Mac, Version 21.0, Armonk. NY: IBM Corp) were used for all analyses.

**Table 2:** Pre- and post-treatment range of motion (ROM) for all treatment groups

	Pre-E/F		Post-E/F		Pre-R/U		Post-R/U		Pre-P/S		Post-P/S	
	Mean	SD	Mean	SD	Mean	SD	Mean	SD	Mean	SD	Mean	SD
STT (*n* = 27)	91°	4.5	83°	16.4	42°	11.6	33°	9.9	167°	13.5	155°	20.2
RSO (*n* = 14)	73°	8.0	93°	42.5	37°	10.1	43°	19.8	166°	10.4	149°	17.2
VBG (*n* = 4)	96°	12.5	70°	55.7	51°	26.6	30°	27.8	165°	19.1	150°	0
RSO and VBG (*n* = 2)	115°	15.0	95°	7.1	55°	7.1	48°	10.6	170°	14.1	145°	7.1
Post-treatment differences												
RSO vs. STT (*P* value)			0.38^a^				0.09^a^				0.48^a^	
Estimated clinically relevant difference			30°^b^				20°^b^				20°	
Sample size calculation			66				34				36	

*STT* scaphotrapeziotrapezoid arthrodesis, *RSO* radial shortening osteotomy, *VBG* vascularized bone graft, *SD* standard deviation

ROM was missing in 4/57 cases. The *t* test was used for statistical analysis. Sample size per group calculation was based on *α* = 0.05, power = 80 %, a two-sided *t* test for independent groups, and a clinically relevant difference of 10 % in the highest score

^a^Wilcoxon test

^b^Estimation based on functional ranges of motion of the wrist [40]

**Table 3:** Functional outcomes and complications in all treatment groups

	NRS at rest		Grip strength dominant^a^		Grip strength non-dominant^b^		DASH		Time to return to work in weeks		No. of complications^c^	MMWS		Same operation^d^
	Median	SD	Mean (%)	SD	Mean (%)	SD	Mean	SD	Mean	SD	*n* (%)	Mean	SD	*n* (%)
STT (*n* = 27)	1	2	85	23	79	14	44	19	4	1	12 (44)	70	7	24 (89)
RSO (*n* = 14)	1	1	87	17	66	9	43	19	4	1	3 (21)	71	9	14 (100)
VBG (*n* = 4)	0	–	99	5	105	–	47	28	2	1	1 (25)	78	10	4 (100)
RSO and VBG (*n* = 2)	3	–	6	–	78	–	54	11	4	1	0	75	14	0
Post-treatment differences														
STT vs. RSO (*P* value)	0.88^e^		0.86^f^		0.19^f^		0.59^f^		0.93^f^		0.19^e^	0.93^f^		0.20^e^
Estimated clinically relevant difference	2.5^g^		20		20		20^h^		2		20	20		
Sample size calculation	24		44		18		32		12		192	10		

*NRS* Numeric Rating Scale for Pain, *DASH* Disabilities of the Arm, Shoulder, and Hand (DASH) score, *MMWS* Modified Mayo Wrist Score, *STT* scaphotrapeziotrapezoid arthrodesis, *RSO* radial shortening osteotomy, *VBG* vascularized bone graft, *SD* standard deviation

^a^When KD affected the dominant side in relation to the non-dominant side

^b^When KD affected the non-dominant side in relation to the dominant side

^c^Additional procedures were considered as complications of the first surgery (no hematoma or infections were observed). In the STT group, 12 denervations were later performed, and in the RSO group 2 denervations and 1 total wrist arthrodesis

^d^“Yes” response to the question about whether they would have the same operation if they had the choice again

^e^Wilcoxon test

^f^ *t* test

^g^Estimation based on clinically important differences in the 0 to 10 Numeric Rating Scale-Pain Intensity

^h^Estimation based on DASH data of non-clinical vs. clinical groups of persons aged 30–49 years (Jester et al. 2010 [41])

**Table 4:** Pre- and post-treatment assessments of radiological parameters evaluating KD progression in all treatment groups

	Pre-Ståhl index		Post-Ståhl index		Pre-Nattrass index		Post-Nattrass index		Pre-radius-scaphoid-angle		Post-radius-scaphoid-angle	
	Median	SD	Median	SD	Median	SD	Median	SD	Median	SD	Median	SD
STT (*n* = 27)	0.37	0.07	0.32	0.08	0.70	0.06	0.69	0.04	59°	6.11	51°	5.35
RSO (*n* = 14)	0.37	0.1	0.32	0.11	0.70	0.06	0.71	0.08	56°	7.71	63°	5.58
VBG (*n* = 4)	0.48	0.1	0.44	0.09	0.69	0.01	0.69	0.01	62°	7.48	64°	8.49
RSO and VBG (*n* = 2)	0.39	0.04	0.30	0.07	0.72	0.04	0.74	0.05	46°	5.66	60°	8.49
Pre- to post-treatment differences												
RSO (*P* value)	0.21^a^				0.67^a^				0.13^a^			
VBG (*P* value)	1.00^a^				0.32^a^				0.06^a^			
RSO and VBG (*P* value)	0.18^a^				0.18^a^				0.18^a^			
Estimated clinically relevant difference	0.1				0.1				8°			
Sample size calculation	42				24				38			

*STT* scaphotrapeziotrapezoid arthrodesis, *RSO* radial shortening osteotomy, *VBG* vascularized bone graft, *SD* standard deviation

^a^Wilcoxon test

## Results

### Clinical outcomes of STT compared to RSO

There were no significant differences between treatment groups with regard to age, gender ratio, dominant vs. non-dominant side affected, and pre-treatment employment status and occupational activity (Table 1). There were no significant differences in post-treatment ROM for E/F, R/U deviation, and P/S between STT arthrodesis and RSO (Table 2). However, there was a significant reduction in R/U deviation following STT arthrodesis and of P/S following RSO or STT arthrodesis. Furthermore, no significant differences were found between the STT and RSO groups regarding pain at rest or activity-induced on the NRS, DASH, the MMWS, grip strength, time to return to work, and the response to the question regarding whether, if given the choice again, the patient would have the same operation (Table 3).

### Radiological outcomes of RSO or VBG and of RSO and VBG

**Fig. 1:**
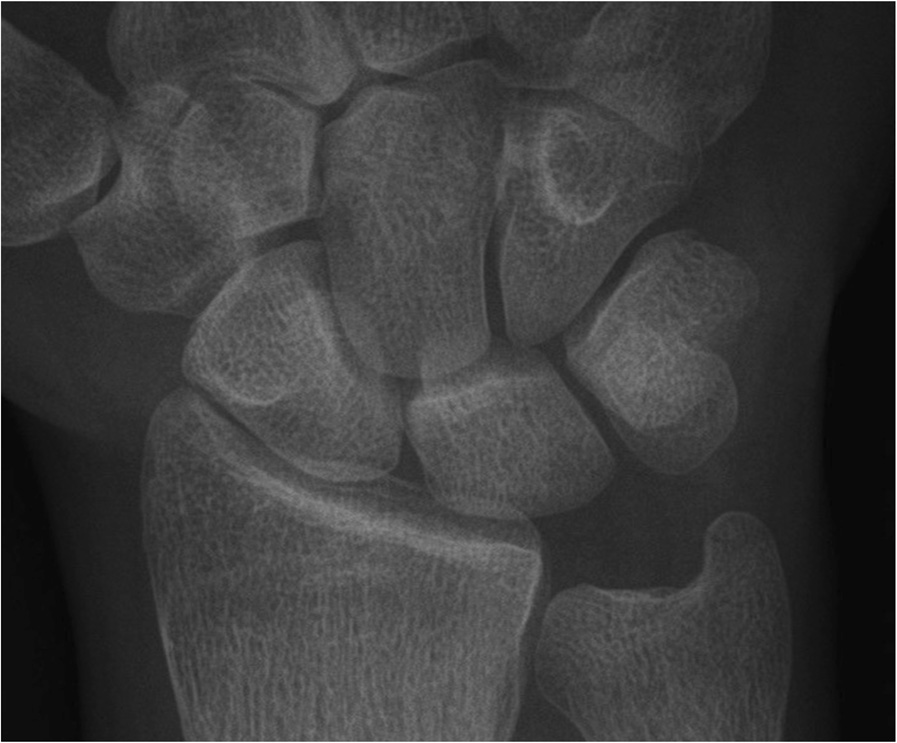
Pre-operative pa x-rays of the right wrist of a 31-year-old male patient who had undergone RSO

**Fig. 2:**
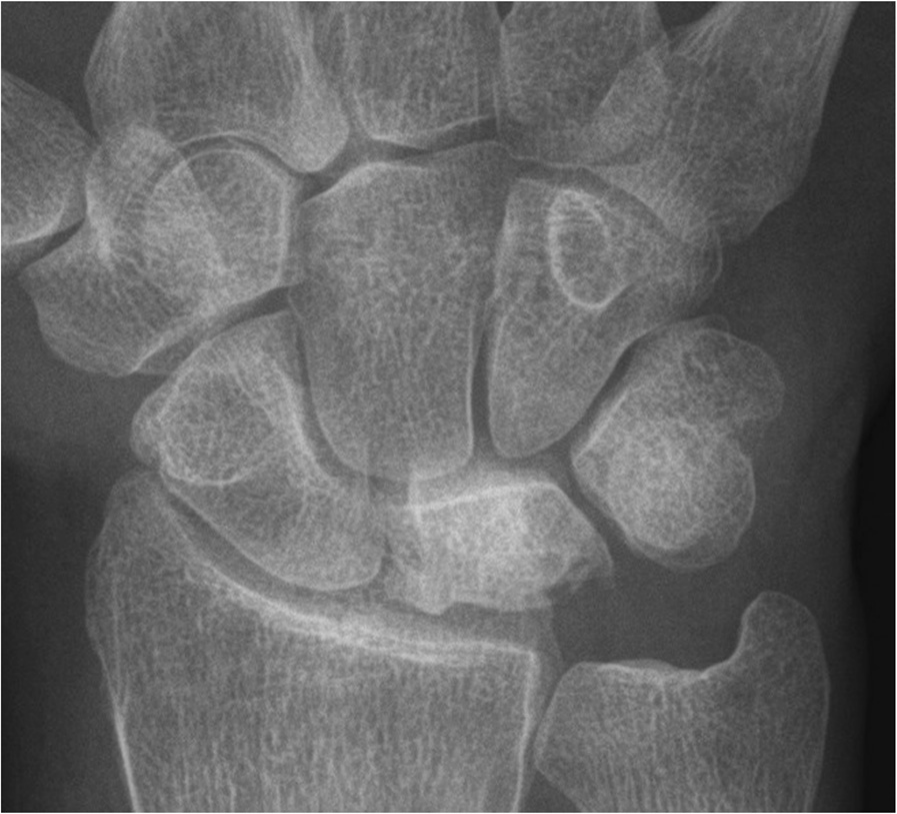
Post-operative pa x-rays of the right wrist of a 31-year-old male patient who had undergone RSO

**Fig. 3:**
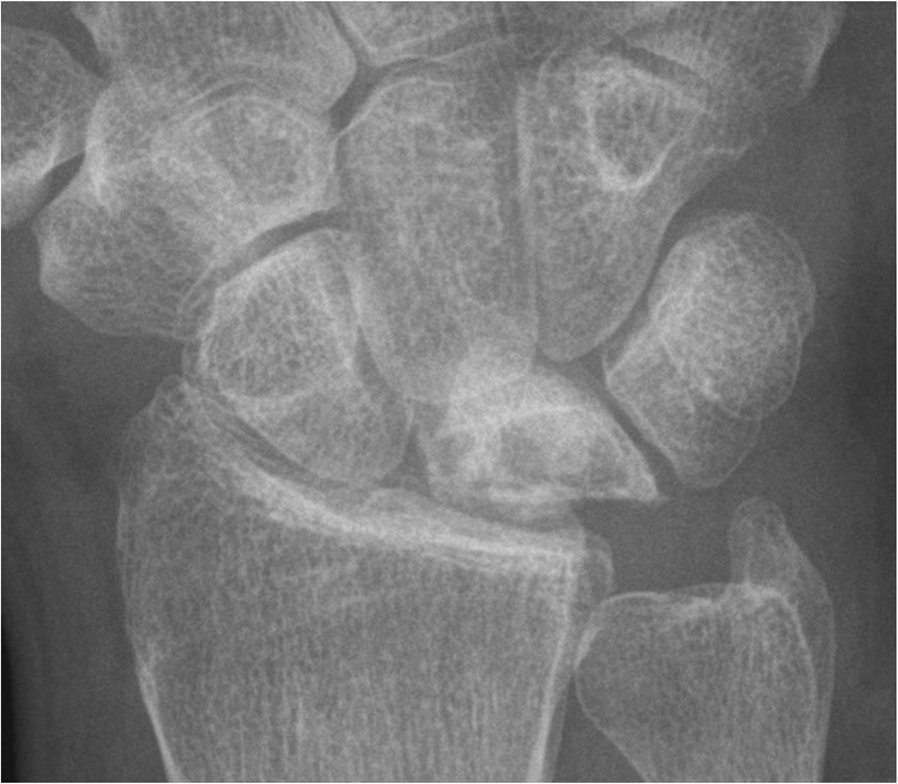
Pre-operative x-rays of the right wrist of of a 45-year-old male patient who had undergone STT arthrodesis

**Fig. 4:**
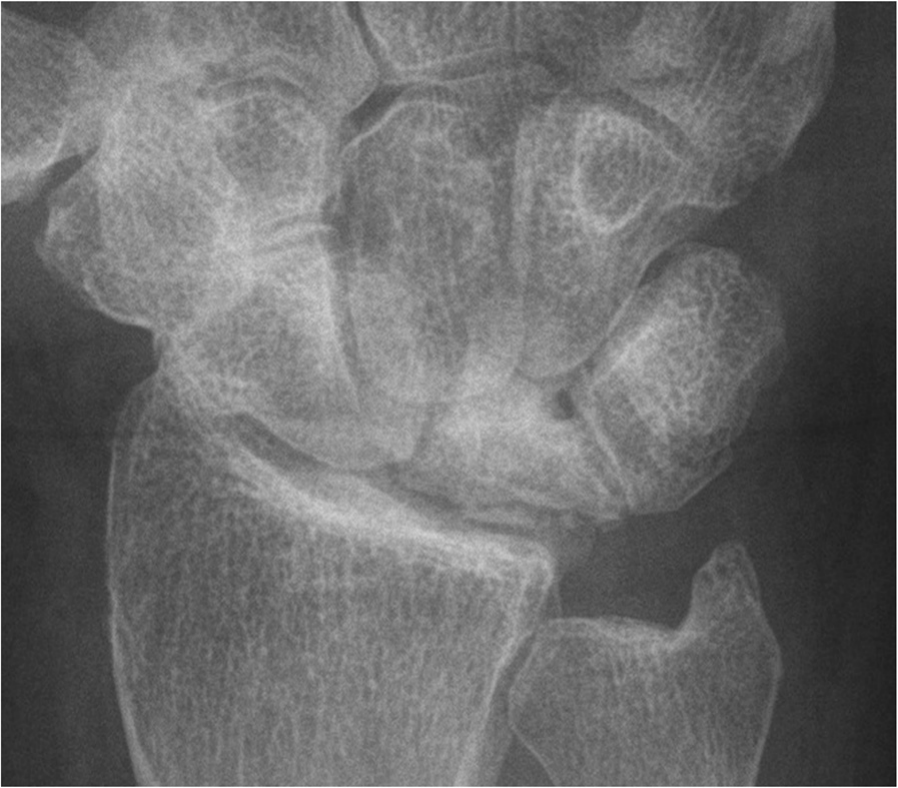
Post-operative x-rays of the right wrist of of a 45-year-old male patient who had undergone STT arthrodesis

**Fig. 5:**
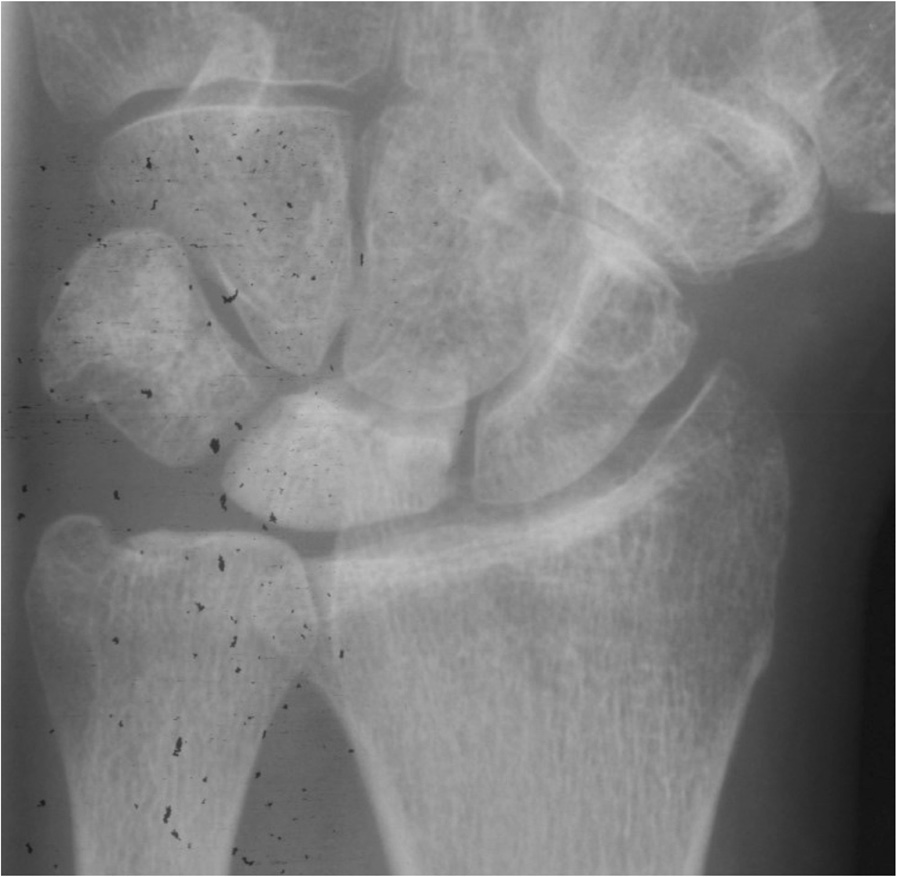
Pre-operative x-rays of the left wrist of of a 44-year-old male patient who had undergone VBG

**Fig. 6:**
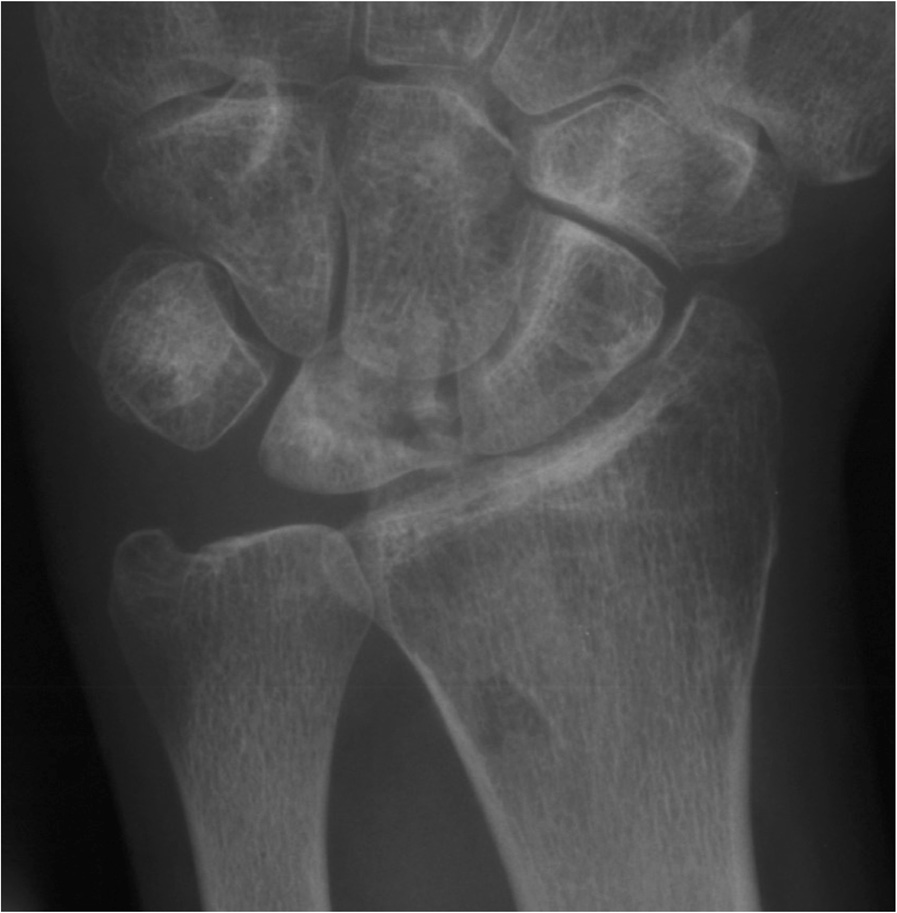
Post-operative x-rays of the left wrist of of a 44-year-old male patient who had undergone VBG

Ståhl index, Nattrass index, and radioscaphoid angle did not significantly change following RSO (Table 4). The mean pre-operative ulnar variance was −2.8 mm, and the mean post-operative ulnar variance was −0.43 mm. Progression of disease stage, according to Lichtman, could be observed in 10/14 patients following RSO, in 2/4 patients following VBG, and in 2/2 patients following RSO combined with VBG (See Figs. 1, 2, 3, 4, 5, 6). There was almost perfect agreement among the three examiners regarding the Ståhl index (lunate height ± 1 mm/lunate width ± 1 mm), Nattrass index (carpal height ± 1 mm/capitate height ± 1 mm), radioscaphoid angle (±5°), and the KD stage (agreement of at least two examiners 48/57 (84 %), 53/57 (93 %), 52/57 (91 %), 55/57 (96 %), respectively (data not shown)).

## Discussion

We compared the clinical outcomes of STT arthrodesis to RSO. In addition, we measured the impact of RSO and vascularized bone grafts (VBG) on disease progression based on X-ray examinations. Of the 57 patients examined, we were unable to recognize clinically relevant differences between RSO and STT arthrodesis or significant radiological differences among RSO, VBG, or RSO combined with VBG.

No evidence beyond expert opinion has been brought forward to determine the indications of STT vs RSO. A US survey has found that most hand surgeons use Lichtman staging and ulnar variance to guide treatment decisions [20]. However, an international survey among hand surgeons has shown that given a stage IIIB according to Lichtman and an ulnar variance of −2 mm, 30 % of the respondents would recommend RSO while 41 % would recommend STT arthrodesis [21]. The divergent opinions on the same case may be explained by the uncertain causal relationship between KD and negative ulnar variance, which questions the validity of ulnar variance for guidance of treatment rational [2]. Furthermore, the prognostic value of the Lichtman classification has been questioned since similar outcomes after RSO have been found in patients with stage II or IIIA (*n* = 17) and IIIB (*n* = 14) [22]. However, numerous other potential indication parameters such as age, handedness, years of active employment, job category, pre-operative range of motion, and grip strength as well as pre-operative Ståhl and Nattrass index and radius-scaphoid-angle were analyzed in order to control for confounding (Tables 1, 2, and 4).

### Strengths and weaknesses of the study

Rigorous criteria limited the inclusion rate but provided adequate data sampling, collection, and analysis thereby improving the quality and validity of our study. To minimize the incidence of false-positive diagnosis of KD, we included only patients with confirmed diagnoses on pre-treatment X-ray examination, CT, and MRI examinations as independently assessed by a board certified plastic surgeon and a musculoskeletal radiologist.

Because KD is not well understood, questions persist as to which parameter is adequate to measure disease progression. The Nattrass and Ståhl indices and the radius-scaphoid-angle have been widely used for radiological evaluation of KD because of good intra-observer reliability [15, 23]. The specificity and sensitivity of these parameters for any change intended by the treatment is unknown although the independent evaluation of these radiological parameters by three hand surgeons minimized observer bias. However, a bias due to inappropriate or imperfect reference standard cannot be ruled out when evaluating the radiological outcome of KD.

Because of a lack of scientific evidence revealed in recent studies for a causal association between KD and negative ulnar variance [2], RSO has not been performed after 2009, leading to an overall longer follow-up. A retrospective study on RSO for KD has suggested that no relevant differences in clinical outcomes occurred in 22 patients at 5 and 10 years follow-up [24]. Our literature review did not suggest a unidirectional change in outcome measures after STT arthrodesis for KD between medium- and long-term periods (Table 6). Nevertheless, the difference in follow-up periods of patients after STT arthrodesis or RSO may have influenced statistical significance. Further studies are needed to determine if clinical changes are significant between medium- and long-term periods and whether or not these differences are clinically relevant for the patient.

Demographic parameters and stage distributions were comparable among all treatment groups except when comparing the pre-operative stages in the RSO group and in the RSO and VBG groups. However, group classification according to the initial stage of KD was not performed for the following reasons: (1) it has been previously suggested that initial KD stage, according to Lichtman, has no influence on treatment outcome [25, 26]; (2) the validity of current classifications has been questioned because arthritis of the lunate can be observed in the absence of lunate fracture or carpal collapse [1]; (3) the reliability and comparability of KD stages in literature is limited because no distinction is generally made regarding the localization of arthritis (lunate cartilage, lunate facet, or the entire radiocarpal joint) and because there is no consensus regarding the diagnostic requirements for KD and associated arthritis (X-ray, MRI, CT scan, or arthroscopy) [1]; (4) because KD may progress from a preserved lunate shape to fragmentation within 6 months [1], staging may not be accurate if more than 4 weeks have elapsed between diagnosis and surgery; (5) a correlation between radiological parameters of KD classifications and the clinical course has not been established, while many authors have observed a poor correlation with clinical findings [27, 28].

Given the retrospective nature of this comparative study, the treatment decisions for STT arthrodesis, RSO, and VBG were not defined in advance but based on previously published expert opinion [10] and reflect the variability in treatment recommendations among hand surgeons [20, 21].

The inability to accrue enough patients to generate high statistical power in clinical trials is a problem common to all rare diseases. Because of this challenge, underpowered but well-conducted large studies provide the best available data until a meta-analysis may be conducted to provide results with higher statistical power. We believe that the descriptive statistics and the review of literature are informative enough to open a debate on the achievable goals in surgical treatment of KD.

### Clinical outcomes

A systematic review of 175 non-comparative case series on KD treatment outcomes showed an increasing trend towards recommending a surgical procedure for KD [5]. However, comparative studies reported comparable results from surgical treatment and, therefore, their conclusions were more cautious [27–33] (Table 5). A meta-analysis suggested that there was insufficient data to determine the superiority of any intervention compared to placebo or the natural history of the disease [4].

**Table 5:** Review of comparative outcome studies with extractable data

Author	Data assessment	Treatment (inclusion rate of wrists with KD^d^)	Follow-up in years (SD)	Post-op pain	Post-op DASH without work module	Post-op E/F	Incapacity for work (weeks)	Significant difference in clinical outcome
Afshar, 2013 [42]	Chart review	RSO (9/9)	6.4 ± 1.8	NM	NM	NM	NM	None
	Examination	VBG (7/7)	6.5 ± 1.6	NM	NM	NM	NM	
Martin, 2013 [43]	DASH only	Conservative (44/44)	NM	NM	23.7 ± 24.5	NM	NM	None
		Partial wrist fusion (11/11)	NM	NM	20.0 ± 20.1	NM	NM	
		Complete wrist fusion (5/5)						
		Lunate excision (1/1)						
		RSO (1/1)						
Hohendorff, 2012 [44]	Chart review	STT (8/8)	1	VAS at rest^b^, 28 ± 31	21 ± 16	56° ± 16	NM	Better E/F and R/U after PRC
				VAS activity-induced^b^, 30 ± 27				
	Examination	PRC (11/11)	1	VAS at rest^b^, 16 ± 29	19 ± 20	80° ± 23	NM	
				VAS activity-induced^b^, 30 ± 26				
Van den Dungen, 2006 [45]	Chart review	Conservative (19/59)	12	Pain quality and duration	21	92°	2.6	Less pain, better ROM, faster return to work after conservative treatment
	Examination	STT (11/25)	14	Pain quality and duration	17	74°	17.1	
Das Gupta, 2003 [46]	Examination	STT (13/13)	1.9	NM	19	68°	NM	NM
		RSO (20/42)	6.9	NM	14	106°	NM	
Salmon, 2000	Chart review	Conservative (15/18)	NM	NRS at rest^b^, 2.8	NM	NM	NM	NM
				NRS at worst^b^, 3				
	Examination	RSO (14/15)	NM	NRS at rest^b^, 0.5	NM	NM	NM	
				NRS at worst^b^, 7.6				
Nakamura, 1998 [48]	Chart review	PRC (7/7)	6.7	Pain yes/no	NM	64°	NM	None
		STT (7/7)	3.5	Pain yes/no	NM	76°	NM	
		SC (4/4)						
		RL (3/3)						
		LC (1/1)						
Delaere, 1998 [49]	Chart review	Conservative (22/22)	5.4	Pain quality and duration	NM	97°	NM	Better ROM after conservative treatment
	Examination	STT (11/11)	5.5	Pain quality and duration	NM	68°	NM	
		PRC (6/6)						
		Vessel implantation (3/3)						
		RSO (1/1)						
		Ulnar lengthening (1/1)						
		Denervation (1/1)						
Condit, 1993 [50]	Chart review	RSO (14/15)	5.2	NM	–	NM	NM	Better clinical outcome after RSO according to own wrist scoring system
	Examination	STT (9/9)	4.5	NM	–	NM	NM	
Kristensen, 1986 [51]	Chart review	Immobilization (23/23)	23	VRS^a^ (0–3), 2 ± 0.7	–	NM	NM	NM
	Examination	No specific treatment (24/24)	18.2	VRS^a^ (0–3), 2 ± 0.7	–	NM	NM	
Evans, 1986 [52]	Chart review	Conservative (14/14)	1.8	VRS^a^ (0–3), 1 ± 0.7	–	68°	NM	NM
	Examination	Silastic arthroplasty (21/21)	3.2	VRS^a^ (0–3), 1 ± 0.8	–	69°	NM	
Beckenbaugh, 1980 [53]	Chart review	Conservative (7/10)	7	No patient had pain	–	89°	NM	None
	Examination	Silastic arthroplasty (22/22)	3.8	No patient had pain	–	75°	NM	
Stahl, 2015 [54]	Chart review	STT (27/46)	4^b^ ± 3^c^	NRS at rest, 0^a^ ±2 ^c^	44.3^b^ ± 19^c^	83°^b^ ± 16^c^	4^b^ ± 3^c^	None
				NRS activity-induced, 5^a^ ±2^c^				
	Questionnaire	RSO (14/21)	10^b^ ± 7.4^c^	NRS at rest, 1^a^ ± 1^c^	43.9^b^ ± 19^c^	93°^b^ ± 42^c^	5^b^ ± 3^c^	
	Examination			NRS activity-induced, 6^a^ ±2 ^c^				

*NRS* Numeric Rating Scale for Pain (0–10), *VAS* visual analog scale (0–100), *VRS* verbal rating scale, *NM* not mentioned, *Δ* Difference between pre- and post-treatment values, *PRC* proximal row carpectomy, *STT* scaphotrapeziotrapezoid arthrodesis, *RSO* radial shortening osteotomy, *VBG* vascularized bone graft

^a^Median

^b^Mean

^c^Standard deviation

^d^Ten out of 12 of the reviewed studies did not mention if the data were collected prospectively in a follow-up examination for the purpose of a clinical study or if the data were assessed on the occasion of clinical follow-up examination and retrospectively analyzed. None of the studies provided a flow chart of patient enrolment with the number of patients excluded at each stage

No significant differences were found in a follow-up examination of 33 conservatively treated patients between stages II, IIIA, IIIB, and IV regarding ROM, DASH score, pain, or grip strength although the DASH score seemed to improve spontaneously in patients who had experienced symptoms for longer than 10 years [28]. The homogenous clinical outcomes of surgically treated patients may be due to partial wrist denervation during the procedures, limited specificity and sensitivity of outcome parameters, the relatively benign spontaneous course, insufficient statistical power, or a placebo effect. A 5-month follow-up examination of 17 patients after arthroscopic débridement and partial wrist denervation reported decreased pain in 11/17 of the patients [34]. Because there was no evidence that arthroscopy cured or arrested osteonecrosis, the effect may have been due to either partial wrist denervation or to a placebo effect, as previously described in arthroscopic surgery [35].

The lack of clinically relevant differences in treatment outcomes following RSO or STT arthrodesis, as shown in our study, compares well with previous comparative studies (Table 5) and the outcomes of these procedures as predicted by 126 surgeons in an international survey in 2009 and 2010 [21]. Although different scales were used, no striking difference regarding post-treatment pain was observed. The scores measured with the official DASH suggested worse functional outcomes in comparison with other comparative studies. However, uncertainty remains regarding whether the DASH cut points were appropriate for different populations at an individual level [36]. Despite its wide usage, the DASH has some limitations. With respect to question No. 18 of the DASH questionnaire, for example, none of the patients in our study could relate to forceful recreational activities associated with golfing, while the comparison of the level of intensity of golf and hammering may seem questionable. In addition, question No. 20 leaves room for interpretation as to whether difficulties in managing transportation need to include biking or public transportation. Due to the heterogeneous measurement of grip strength in the reviewed literature, no comparisons were possible.

### Radiological outcomes

Differences of 3 to 6 % between pre- and post-operative radiological measurements are of questionable relevance (Table 6). Interestingly, the few studies on RSO with clinical and radiological assessment reported satisfactory clinical results in the face of KD progression upon X-ray evaluation [26, 37, 38]. Relevant and significant differences have not been reported in comparative studies. In an uncontrolled evaluation of one radiologist after VBG, suggesting revascularization in 12/17 cases on T1-weighted and/or T2-weighted MRI, no efforts were made to reduce the risk of bias [39]. Indeed, vaguely defined radiological parameters of unknown reliability and measurements of one single unblended observer are prone to an observer-expectancy effect.

**Table 6:** Review of radiological outcomes after RSO and/or VBG studies with extractable data

	Treatment (inclusion rate of wrists with KD)	Follow-up in years (min, max)	Δ Ståhl index	Δ Carpal height index	Δ Radioscaphoid angle	Significant difference in radiological outcome of comparative studies
Matsui, 2014 [55]	RSO (11/11)	14.3	+0.01	0	NM	–
Fujiwara, 2013 [56]	VBG'd' alone or combined with capitate shortening or RSO (8/8)	NM	+0.03^b^	+0.028^b^	NM	NM
	VBG (10/10)	NM	−0.004	+0.004	NM	
Watanabe, 2008 [57]	RSO (13)	21	−0.03^b^	−0.01^b^	−3°	–
Wada, 2002 [58]	RSO (13)	14	−0.06^b^	−0.03^b^	NM	–
Iwasaki, 2002 [59]	Closing wedge osteotomy (11)	2.25	−0.06	−0.03	+5.6°^b^	No
	RSO (9)	2.6	0	+0.01	−1.1	
Moran, 2002 [60]	VBG (26)	2.6	0.02^c^	0.008^c^	NM	–
Wintman, 2001 [61]	RSO (72/88)	2.6 ± 2.2	NM	+0.01^b^	NM	–
Salmon, 2000 [47]	Conservative (15/18)	NM	Δ carpal height, −0.7 mm	NM	+5.4°	NM
			Δ lunate width, +2.0 mm			
	RSO (14/15)	NM	Δ carpal height, −0.7 mm	NM	+2.1°	
			Δ lunate width, +2.4 mm			
Stahl, 2015 [54]	RSO (14/21)	10.5 ± 7.4	−0.03	−0.01^a^	+7°	No
	VBG (4/7)	1.2 ± 0.7	−0.04	0^a^	+2°	
	VBG and RSO (2/3)	3.9 ± 1.1	−0.09	+0.02^a^	+14°	

*Δ*difference between pre- and post-treatment values, *NM* not mentioned, *STT* scaphotrapeziotrapezoid arthrodesis, *RSO* radial shortening osteotomy, *VBG* vascularized bone graft

^a^Nattrass Index

^b^ *P* < 0.05 in pre- and post-treatment comparison

^c^No specification if pre-treatment values increased or decreased by the cited figures

^d^Combined with cancellous bone graft

The sample sizes of VBG (*n* = 4) and VBG and RSO (*n* = 2) allow for a descriptive analysis but not the testing of statistical hypotheses. Statistical comparison of clinical outcome parameters was therefore performed between STT (*n* = 27), which aims to prolong wrist function, and RSO only (*n* = 14). However, because the radiologic parameters in this study and in the reviewed literature did not suggest a relevant improvement of KD after RSO or VBG, an improvement of wrist function seems unlikely even in a larger cohort.

## Conclusions

No evidence has been found that the progression of KD can be stopped or reversed by either RSO or VBG. No relevant clinical differences were found following STT arthrodesis or RSO in patients with KD. This study has shown that comparative studies are very rare and that most report negative results. A systematic review has shown that the vast majority of publications on KD are case series, most reporting positive results [5]. Since funding is not easily obtained for research on rare diseases, and even less for non-life threatening diseases, randomized controlled trials are unlikely to be conducted in the near future. In the absence of randomized controlled trials, surgeons may be particularly vulnerable to a bias towards publishing positive results in case series of KD.

### Ethical approval

The study was conducted with the approval of the Ethics Review Board of Eberhard-Karls-University, Tuebingen, Germany (approval number 176/2009BO1).
